# Depletion of Host and Viral Sphingomyelin Impairs Influenza Virus Infection

**DOI:** 10.3389/fmicb.2020.00612

**Published:** 2020-04-30

**Authors:** Amani Audi, Nadia Soudani, Ghassan Dbaibo, Hassan Zaraket

**Affiliations:** ^1^Department of Experimental Pathology, Immunology and Microbiology, Faculty of Medicine, American University of Beirut, Beirut, Lebanon; ^2^Center for Infectious Diseases Research, Faculty of Medicine, American University of Beirut, Beirut, Lebanon; ^3^Doctoral School of Science and Technology, Research Platform for Environmental Science (PRASE), Faculty of Sciences, Lebanese University, Beirut, Lebanon; ^4^Department of Pediatrics and Adolescent Medicine, Faculty of Medicine, American University of Beirut Medical Center, Beirut, Lebanon; ^5^Department of Biochemistry and Molecular Genetics, Faculty of Medicine, American University of Beirut, Beirut, Lebanon

**Keywords:** influenza virus, lipid rafts, sphingomyelin, acid sphingomyelinase, viral envelope, plasma membrane, bacterial sphingomyelinase

## Abstract

Influenza A virus (IAV) is a major human respiratory pathogen causing annual epidemics as well as periodic pandemics. A complete understanding of the virus pathogenesis and host factors involved in the viral lifecycle is crucial for developing novel therapeutic approaches. Sphingomyelin (SM) is the most abundant membrane sphingolipid. It preferentially associates with cholesterol to form distinct domains named lipid rafts. Sphingomyelinases, including acid sphingomyelinase (ASMase), catalyzes the hydrolysis of membrane SM and consequently transform lipid rafts into ceramide-enriched membrane platforms. In this study, we investigated the effect of SM hydrolysis on IAV propagation. Depleting plasma membrane SM by exogenous bacterial SMase (bSMase) impaired virus infection and reduced virus entry, whereas exogenous SM enhanced infection. Moreover, the depletion of virus envelope SM also reduced virus infectivity and impaired its attachment and internalization. Nonetheless, inhibition of ASMase by desipramine did not affect IAV infection. Similarly, virus replication was not impaired in Niemann-Pick disease type A (NPA) cells, which lack functional ASMase. IAV infection in A549 cells was associated with suppression of ASMase activity starting at 6 h post-infection. Our data reveals that intact cellular and viral envelope SM is required for efficient IAV infection. Therefore, SM metabolism can be a potential target for therapeutic intervention against influenza virus infection.

## Introduction

Influenza is considered a major public health concern due to the significant morbidity and mortality associated with the disease (Lee et al., 2018). Recent studies showed that respiratory infections-associated with seasonal influenza viruses are responsible for 290,000 to 650,000 deaths per year (Lee et al., 2018). Influenza viruses are negative-sense, single-stranded, segmented RNA viruses that belong to the *Orthomyxoviridae* family. The virus is made up of a viral envelope, a matrix layer, and a central core with 8 RNA segments that encode for at least 11 functionally important proteins required during the virus replication cycle (Klumpp et al., 1997; Coloma et al., 2009; Zheng and Tao, 2013).

During its infectious cycle, influenza A virus (IAV) has to cross the plasma membrane during entry and budding from host cells. Therefore, the membrane properties and integrity are important determinants of efficient infection. IAV has been shown to selectively bind to host membrane lipid rafts (Eierhoff et al., 2010; Verma et al., 2018). These specialized membrane microdomains are formed from the preferential association of cholesterol with sphingolipids (Zhang et al., 2009). Additionally, sphingolipids have been implicated during different aspects of the viral life cycles including attachment (Puri et al., 2004; Rawat et al., 2004; Grassme, 2005), entry (Nieva et al., 1994; Miller et al., 2012; Shivanna et al., 2015; Drake et al., 2017), replication (Weng et al., 2010; Martín-Acebes et al., 2016) and budding (Nguyen and Hildreth, 2000; Ono and Freed, 2001; Tafesse et al., 2013). As such, they are considered a promising therapeutic target against viral infections (Yager and Konan, 2019). Several studies have demonstrated a pivotal role for sphingolipids in regulating IAV life cycle. Sphingosine-1-phosphate- (S1P) metabolizing enzymes have been shown to modulate influenza infection *in vitro* and *in vivo* (Seo et al., 2010, 2013; Xia et al., 2018). Sphingosine kinase 1 (SK1) enhances viral replication through regulating viral RNA synthesis and export of nuclear viral ribonucleoprotein complex (Seo et al., 2013). Glucosylceramidase is critical during infection by controlling the successful trafficking of influenza virus to the late endosome and its subsequent fusion and entry (Drews et al., 2019). We have previously demonstrated that *de novo* ceramide plays a protective antiviral role against IAV infection (Soudani et al., 2019). Furthermore, exogenous short-chained ceramide enhances the maturation and activation of dendritic cells in response to IAV infection, thus blocking its replication (Pritzl et al., 2015). Therefore, the sphingolipid biosynthesis is a promising host target for developing novel therapeutic approaches against influenza infection.

Sphingomyelin (SM) is the most abundant membrane sphingolipid. It is predominantly found in the outer leaflet of the plasma membrane, endomembranes, as well as in the intracellular organelles (Slotte, 2013). Depletion of host membrane cholesterol using Methyl-β-Cyclodextrin (MβCD) reduced IAV binding and internalization (Eierhoff et al., 2010; Verma et al., 2018). However, MβCD also depletes SM, whose effect on IAV binding and internalization has been overlooked. The level of cellular SM is primarily regulated by sphingomyelinases (SMases), which catalyze its hydrolysis back into ceramide and phosphorylcholine (Goñi and Alonso, 2002; Hannun and Obeid, 2018). SMases are classified based on their pH optimum into acid (ASMase), neutral (NSMase), and alkaline (AlkSMase) sphingomyelinases (Goñi and Alonso, 2002). Among these, the lysosomal ASMase is the best characterized and is mainly responsible for membrane SM turnover (Goñi and Alonso, 2002; Hannun and Obeid, 2018). Lysosomal ASMase is translocated to the outer leaflet of the plasma membrane by exocytosis, where it catalyzes the hydrolysis of membrane SM and leads to the formation of ceramide-enriched membrane platforms (Zhang et al., 2009; Hannun and Obeid, 2018).

Sphingomyelin and/or ASMase were shown to affect the infectivity of several viruses. SM and its hydrolyzing enzyme ASMase were implicated during Ebola virus infection (Miller et al., 2012). Recently, alphaherpesviruses were shown to exhibit a variable requirement for SM and ASMase during entry into host cells (Pastenkos et al., 2018). Japanese encephalitis virus attachment and entry into target cells was shown to be dependent on SM synthase 1-generated SM (Taniguchi et al., 2016). It was also reported that alphaviruses require membrane sphingolipids, specifically SM, as a cofactor for virus fusion (Nieva et al., 1994). Moreover, Rubella virus was shown to directly bind to SM and cholesterol/SM-enriched microdomains (Otsuki et al., 2018). Lipidomic analyses have shown an increase in the SM content of IAV-infected cells (Tanner et al., 2014). In addition, it was also demonstrated that the SM biosynthetic pathway is important for the transport of influenza virus glycoproteins to lipid rafts located at the apical surface of the plasma membrane (Tafesse et al., 2013).

Here, we investigated the role of SM and it hydrolyzing ASMase and its substrate SM in IAV infection. We found that genetic deficiency or pharmacological inhibition of ASMase does not prevent IAV infection. However, IAV infection was attenuated by the depletion of membrane SM and enhanced upon treatment with exogenous SM. SM depletion of the plasma membrane or the viral envelope impaired IAV attachment and internalization. These results demonstrate that IAV does not require ASMase activity for infection and that intact SM in both cell membrane and viral envelope is essential for efficient IAV entry.

## Materials and Methods

### Cells and Viruses

Madin-Darby canine kidney cells (MDCK) were obtained from Biodefense and Emerging Infections Research Resources Repository (BEI Resources), human alveolar lung carcinoma (A549) were obtained from ATCC (Manassas, VA, United States), healthy primary human fibroblasts (GM00038) and Niemann-Pick disease type A (NPA) fibroblasts (GM00112 and GM13205) were from Coriell Cell Repository (Camden, NJ, United States). Cells were grown in Dulbecco’s Modified Eagle Medium (DMEM) supplemented with 1% penicillin/streptomycin (P/S) and 10% fetal bovine serum (FBS). GM00038 cells were supplemented with 15% FBS. GM13205 cells were supplemented with 1% sodium pyruvate in addition to the previously mentioned supplements. All cells were maintained at 37°C in a humidified incubator containing 5% CO_2_.

A/Puerto Rico/8/1934 (PR8) H1N1 virus was obtained from St. Jude Children’s Research Hospital repository and A/California/07/09 (Cal07) H1N1pmd09 virus was obtained from BEI Resources. The viruses were propagated on MDCK cells supplemented with viral infection medium (VIM) consisting of 1X minimal essential media (10X MEM) with 0.3% (vol/vol) bovine serum albumin (BSA; 10% solution), 0.22% sodium bicarbonate (NaHCO3; 7.5% solution), 1% MEM vitamin (100X solution), 1% glutamax (100X), 1% penicillin-streptomycin (10,000 units penicillin and 10,000 units streptomycin per ml) and 0.08% gentamicin (50 mg/ml). VIM was supplemented with 1 μg/ml of L-1-tosylamido-2-phenylethyl chloromethyl ketone (TPCK)-treated trypsin (Sigma) just before addition to the infected MDCK cells.

### Virus Infections

A549 cells seeded in a 12-well tissue culture plate at 3.2 × 10^5^ cells/well were washed two times with phosphate buffer saline with calcium and magnesium (PBS^+^) and incubated with VIM in the presence or absence of the tested drug. The cells were then infected with 200 μl of the virus diluted in VIM in the presence or absence of the drug at 37°C for 1 h. Afterward, cells were washed twice with mild acidic PBS^+^ (pH 5) to remove unbound viral particles. Subsequently, cells were incubated with pre-warmed VIM containing 0.2 μg/ml TPCK-treated trypsin (Sigma) at 37°C in the presence or absence of the drug and the virus yield was determined by plaque assay as described below.

### Reagent Preparation

A 10 mM of desipramine (Sigma, D3900) stock, an inhibitor of ASMase (Hurwitz et al., 2009), was prepared in sterile distilled water, stored at −80°C and diluted in VIM just before use. SM (Santa Cruz Biotechnology, CAS 85187-10-6) was dissolved in 100% ethanol, stored at −20°C, and diluted in VIM before use (Martín-Acebes et al., 2016). *Staphylococcus aureus* SMase (ENZO Life Sciences), BML-SE108-0050) was diluted in Hanks balanced salt solution with calcium and magnesium (HBSS^+^) immediately before adding to cells.

### Cytotoxicity Assay

The viability of cells after concentration or time-dependent treatments of drugs was determined using the standard MTT [3-(4,5-dimethyl-2-thiazolyl)-2,5-diphenyl-2H-tetrazolium bromide] assay. All the treatments were done using 2 × 10^4^ cells/well in 96-well tissue culture plates. Briefly, A549 cells were treated with increasing concentrations of the tested drug for a specific period. After incubation, 20 μl of MTT reagent (1 mg/ml in PBS) were added to each well and incubated for 2 h at 37°C. The supernatant was then removed, and the purple formazan crystals were dissolved in 100 μl of solubilizing reagent (acidic isopropanol). Finally, the absorbance was recorded using the Multiskan ELISA reader at 570 nm.

### Plaque Assay

Virus titers were determined using plaque assay in MDCK cells, as previously described (Huprikar and Rabinowitz, 1980). Briefly, 10-fold serial dilutions from the virus collected from treated or untreated A549 cells were prepared. The confluent monolayers of MDCK cells, in 6-well tissue culture plates, were infected with 200 μl of the virus and incubated at 37°C for 1 h, with gentle shaking every 15 min. Then, the virus was removed, and the monolayers were covered with a nutritive medium containing 0.5% agarose and 1 μg/ml TPCK-trypsin. After incubation at 37°C for 72 h, the agar overlay was removed, and the cells were stained with crystal violet containing paraformaldehyde. Finally, the plaques were counted, and the virus titer was calculated.

### ASMase Activity Assay

A549 cells were scraped from the culture plates, centrifuged, washed with ice-cold PBS, and pelleted by centrifugation at 800 *g* for 10 min. Cells were re-suspended in 100 μl ASMase acidic lysis buffer (50 mM sodium acetate, pH 5.0, 1% Triton X-100, 1 mM EDTA) and incubated for 1 h at 4°C (vortexing every 30 min). Cell lysates were then subjected to centrifugation at 14,000 *g* at 4°C for 10 min. The supernatant was isolated and an aliquot containing 30 μg protein (determined by DCTM Protein Assay Kit from BIO-RAD). The sample protein was analyzed for ASMase-activity using Amplex^®^ Red Sphingomyelinase Assay Kit (Invitrogen, A12220) using the two-step process based on the manufacturer’s instructions.

### Total Viral RNA Quantification by RT-PCR

The supernatant of bacterial SMase (bSMase)-treated and untreated cells was collected, followed by total viral RNA extraction using PureLink Viral RNA/DNA Minikit (Invitrogen) according to the manufacturer’s protocol. Quantitative RT-PCR was performed using primers and probes targeting the M gene (IAV) (World Health Organization, 2018). One-Step RT-PCR AgPath-ID (Thermo Fisher Scientific) was used to amplify the target according to the following protocol: 45°C for 10 min, 95°C for 10 min, and then 45 cycles of 95°C for 15 s and 55°C for 1 min. In each sample, the viral gene copy number was assessed using a standard curve generated from AMPLIRUN Influenza A H1 RNA Control (Vircell). The results were plotted in terms of fold-change relative to the control.

### Sphingomyelin Depletion Assay

A549 cells were plated on the day prior to treatment and incubated overnight at 37°C. The cells were incubated with increasing bSMase concentrations (0.1, 0.5, and 1 U/ml) at 37°C for 2 h and then washed twice with HBSS^+^ before infection with either PR8 or Cal07. Virus yields in bSMase-treated and untreated cells were determined using plaque assay and RT-PCR as described above.

For depletion of viral envelope SM, PR8 high titer stock was mixed with the vehicle (HBSS^+^) or 0.1 U/ml bSMase and incubated at 37°C for 1 h. A549 cells were infected with 1 MOI of the treated or untreated IAV (PR8). Virus yield was then quantified at 24 hpi using the plaque assay. Alternatively, the infectivity of SM-depleted viruses was directly assessed using the plaque reduction assay (Hsieh et al., 2016). Briefly, MDCK cells (8 × 10^5^ cell/well) were seeded in six-well tissue culture plates 1 day before infection. The cells were inoculated with PR8 at 90 PFU/well and kept on ice for 1 h, followed by incubation for 20 min at 37°C. Then the cells were washed with acidic PBS^+^ and neutralized with neutral PBS^+^. An overlay of 0.5% nutritive agarose was added to the cells and incubated at 37°C for 72 h. The overlay was then removed and the cells were stained with crystal violet. The plaques were then counted, and the percent reduction in infectivity was calculated.

### Immunofluorescence Assays

A549 cells were fixed in freshly prepared 4% paraformaldehyde for 15 min at room temperature. For ceramide staining, the cells were left unpermeabilized. However, to visualize the internalized influenza virus particles, the cells were permeabilized using 0.2% Triton X-100 at room temperature for 20 min. The cells were blocked with bovine serum albumin (1% BSA in PBS) for 1 h and incubated with either mouse anti-ceramide antibody (Sigma, C8104-50TST), mouse anti-nucleoprotein (NP) antibody (BEI Resources, NR19868) or mouse anti-influenza A (H1N1 antigen) antibody (Merck Millipore, MAB8256) at 1:100 in 1% BSA overnight at 4°C. The cells were rinsed with PBS and incubated for 1 h with Alexa Flour 488 goat anti-mouse secondary antibody (Thermofisher Scientific, A11001) at 1:250 in 1% BSA. Coverslips were mounted with a fluoroshield mounting medium containing DAPI. The images were acquired with a 40x or 63x oil objective lens, as indicated in the figure legends using a Zeiss LSM 710 laser-scanning confocal immunofluorescence microscopy. Quantification of fluorescent intensities was done using ZEN software.

### Attachment and Internalization Assays

A549 cells were incubated with bSMase (1 U/ml), or the vehicle (HBSS^+^) for 2 h at 37°C, and washed twice with HBSS^+^. Treated and untreated cells were preincubated for 15 min at 4°C, after which PR8 15 MOI was inoculated onto the cells and incubated 1 h at 4°C to allow virus adsorption and restrain entry. The cells were then washed twice with cold PBS^+^ and fixed. The attached virions were labeled using an anti-influenza A (H1N1) antibody and quantified using confocal microscopy.

To measure virus internalization, following virus adsorption for 1 h at 4°C, the cells were placed for 30 min at 37°C to allow entry of the adsorbed virions. The cells were then fixed, permeabilized, and assayed by confocal microscopy using an anti-NP antibody.

For assessing attachment and entry of bSMase-treated IAV, PR8 high titer stock was mixed with the vehicle (HBSS^+^) or 0.1 U/ml bSMase and incubated at 37°C for 1 h. The bSMase-treated virus was then used to infect A549 cells as described above.

### Hemagglutination Assay

Hemagglutination assay was done as previously described (Killian, 2014). Briefly, chicken blood was washed up to three times with PBS. The red blood cells were then resuspended in PBS to reach a concentration of 1%. Two-fold virus dilutions were prepared in PBS in 96-well round-bottom culture plate in which 50 μl of the virus was left in each well. Then, 50 μl of the red blood cell suspension was added to each well and left to settle for 30 to 45 min at room temperature before reading.

### Statistical Analysis

All data are represented as means with standard deviations (SD). Statistical analysis was conducted using one-tailed Student’s *t-*test. All statistical tests were performed with Microsoft excel. A *P-*value of less than 0.05 was considered statistically significant.

## Results

### SM Depletion Restricts IAV Replication in A549 Cells

**FIGURE 1:**
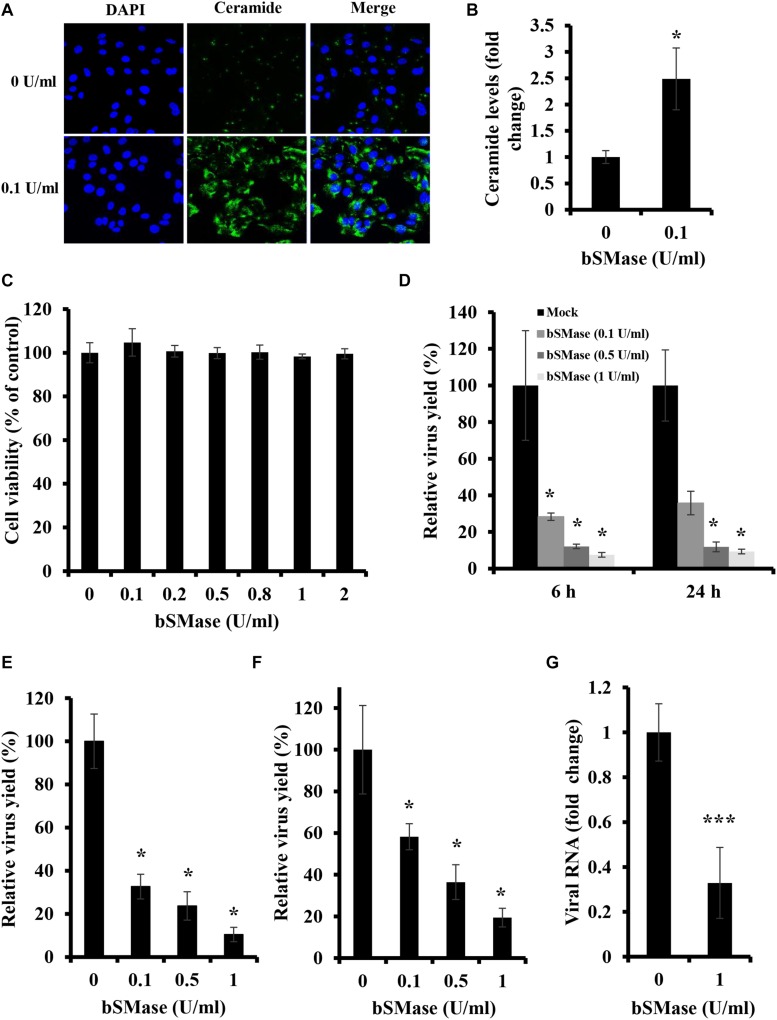
Plasma membrane SM depletion suppresses IAV replication *in vitro*. **(A,B)** The effect of exogenous bSMase on ceramide accumulation was studied using confocal microscopy. A549 cells were treated with 0.1 U/ml bSMase or with the vehicle (HBSS^+^) for 2 h at 37°C. Cells were then washed, fixed, and stained with mouse anti-ceramide antibody followed by Alexa Fluor 488 conjugated anti-mouse antibody (green). The cell nuclei were stained with DAPI (blue). The images were taken using confocal microscopy with a 40x oil objective lens **(A)**. Fluorescent intensities of confocal images were measured, as shown in **(B)** using ZEN software. **(C)** Cytotoxicity of exogenous bSMase on A549 cells was assessed using the MTT assay. A549 cells were treated with increasing concentrations of bSMase for 2 h, washed with PBS^+,^ and incubated with VIM for 24 h. MTT assay was then performed at 24 h post-treatment to determine cell viability in treated and untreated cells. **(D–G)** The effect of SM depletion on IAV infection was determined by the plaque assay **(D–F)** or RT-PCR **(G)**. A549 cells were pretreated with increasing bSMase concentrations or vehicle for 2 h followed by infection with either PR8 at 1 MOI **(D,G)** and 0.01 MOI **(E)** or Cal07 at 1 MOI **(F)**. Viral titers were then determined using the plaque assay at the indicated time points **(D)** or at 24 hpi **(E,F)** and plotted relative to the vehicle-treated cells. Variation in PR8 total viral RNA in the supernatant of bSMase-treated and untreated cells was assessed at 24 hpi by RT-PCR using One-Step RT-PCR AgPath-ID **(G)** and plotted relative to the vehicle-treated cells. The results shown are representatives of two independent experiments. Means ± SD are shown. Statistical significance between untreated and bSMase-treated cells was assessed using the *t*-test (^∗^corresponds to a *P-*value < 0.05, and ^∗∗∗^corresponds to a *P-*value < 0.001).

To assess whether manipulation of host cellular SM affects IAV infection, we depleted membrane SM using exogenous bSMase before infection. Exogenous bSMase specifically hydrolyzes outer membrane SM into phosphorylcholine and ceramide, thus decreasing SM content and increasing ceramide in the outer plasma membrane. Using confocal microscopy and specific ceramide antibody, we demonstrated that bSMase (0.1 U/ml) treatment efficiently increases the plasma membrane ceramide content, verifying efficient hydrolysis of SM (Figures 1A,B). The effect on cell viability of bSMase treatment up to 2 U/ml for 2 h was assessed at 24 h after removing the enzyme, and no cytotoxicity was observed in any of the concentrations used (Figure 1C).

To examine whether the depletion of cellular SM affects IAV propagation, A549 cells were pretreated with increasing bSMase concentrations (0.1–1 U/ml) for 2 h at 37°C, washed to remove bSMase and inoculated with PR8 0.01 MOI. The virus yield was significantly reduced at 24 hpi in SM-depleted cells (Figure 1E). Even when an MOI of 1 was used, virus yield decreased by more than 80% in cells treated with 0.5 and 1 U/ml bSMase at 6 and 24 hpi (Figure 1D).

To verify whether the suppression in virus infection by SM depletion is specific for IAV PR8, we examined the effect of bSMase treatment on the infection with a contemporary A/H1N1pdm09 strain (Cal07). A549 cells were treated with increasing concentrations of bSMase and inoculated with 1 MOI of Cal07. The virus yield was assessed at 24 hpi relative to the control (untreated cells). Similar to PR8, Cal07 virus production was significantly suppressed in bSMase-treated cells in a dose-dependent manner (Figure 1F).

To prove that the reduction in IAV observed in bSMase-treated cells using plaque assay was due to a decrease in virus yield but not the infectivity of the virus propagated in these cells, we quantified the total viral RNA in the supernatant of bSMase-treated (1 U/ml) and untreated cells at 24 hpi. Similar to the results obtained by using plaque assay, the total viral RNA was significantly reduced in the supernatant of bSMase-treated cells compared to the untreated ones (Figure 1G). This finding suggested that that virus yield was actually reduced and not its infectivity.

### Exogenous SM Enhances IAV Production

**FIGURE 2:**
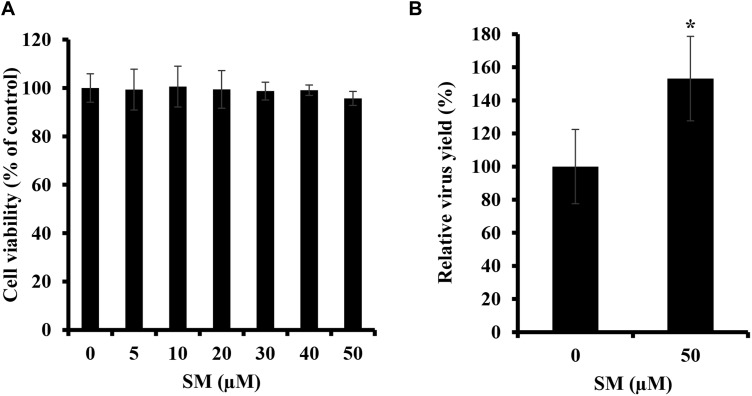
Exogenous SM enhances IAV replication. **(A)** Cytotoxicity of SM on A549 cells was assessed using MTT assay. A549 cells were treated with increasing SM concentrations up to 50 μM, and MTT assay was then performed at 24 h post-treatment to determine cell viability in treated and untreated cells. **(B)** The effect of SM on IAV infection was studied. A549 cells were pretreated with 50 μM of exogenous SM or solvent for 24 h and inoculated with PR8 at 0.01 MOI. Virus titers were then assessed at 24 hpi using the plaque assay and plotted relative to vehicle-treated cells. Means ± SD are shown. Results shown are representative of two independent experiments. Statistical significance was assessed using the *t*-test (^∗^corresponds to a *P-*value < 0.01).

Because the treatment of A549 cells with bSMase suppressed IAV infection, we were interested in determining the effect of enriching A549 cells with exogenous SM on IAV infection. SM used in this study has long fatty acyl chain moiety, which favors its incorporation into the plasma membrane rather than being absorbed into the cells. First, we showed that SM treatments up to 50 μM for 24 h have no cytotoxic effect on A549 cells (Figure 2A). Next, A549 cells were pretreated with 50 μM of SM for 24 h at 37°C. SM was then removed, and cells were washed with PBS^+^ prior to inoculation with 0.01 MOI of PR8, and virus production was assessed in treated and untreated cells at 24 hpi using plaque assay. SM treatment resulted in a moderate but significant increase in virus yield (Figure 2B). This finding shows that SM in the host cells promotes IAV replication.

### SM Is Required for Entry of IAV Into the Host Cell

**FIGURE 3:**
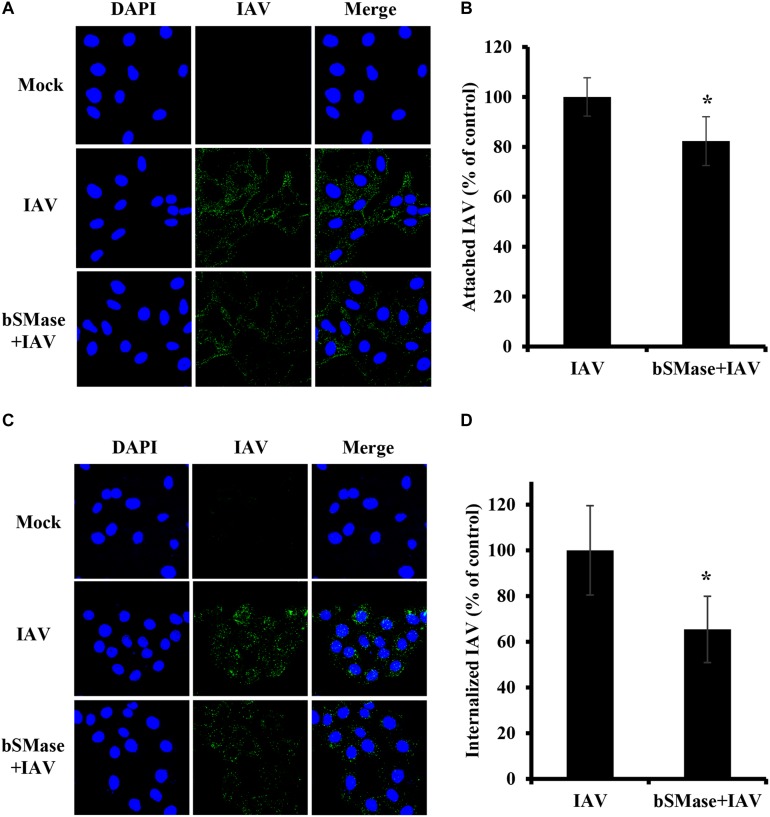
Cellular SM depletion restricts IAV entry into A549 cells. A549 cells were pretreated with 1 U/ml bSMase (bSMase + IAV) for 2 h at 4°C or left untreated (IAV) prior to inoculation with PR8 at MOI of 15. Inoculated cells were kept at 4°C for 1 h to allow virus adsorption and then washed with cold PBS^+^ **(A–D)**. The attached virus was labeled with mouse anti-influenza A (H1N1) antibody followed by Alexa Fluor 488 conjugated anti-mouse antibody (green) **(A,B)**. Alternatively, after viral adsorption at 4°C for 1 h, the cells were placed at 37°C for an additional 30 min to allow virus entry, and internalized virions were assessed using mouse anti-NP antibody followed by Alexa Fluor 488 conjugated anti-mouse antibody (green) **(C,D)**. Mock cells were left untreated/uninfected. The cell nuclei were stained with DAPI (blue). The images were taken using confocal microscopy with a 63x oil objective lens **(A,C)**. The variation of fluorescence intensities was measured using ZEN software **(B,D)**. Means ± SD are shown. Results are representative of two independent experiments. Statistical significance between infected (IAV) and treated infected (bSMase + IAV) cells was assessed using the *t*-test (*corresponds to a *P-*value < 0.05).

The finding that IAV is sensitive to SM levels at the plasma membrane, led us to hypothesize that SM is essential for early steps in the viral life cycle. Therefore, we assessed IAV attachment and internalization upon SM depletion. IAV attachment and internalization were examined by confocal microscopy using anti-influenza A (H1N1) antibody and anti-NP antibody, respectively. We pretreated A549 cells with 1 U/ml bSMase for 2 h at 37°C and infected them with 15 MOI of IAV PR8 on ice for 1 h to allow virus adsorption and block entry. For attachment assay, A549 cells were washed twice with cold PBS^+^ to remove non-adsorbed virions and then immediately fixed and labeled with anti-influenza A (H1N1) antibody. SM depletion resulted in a slight, albeit significant, reduction in virus attachment, suggesting that SM depletion has a minimal effect on IAV attachment (Figures 3A,B).

Next, we examined whether SM is required for virus internalization. The cells were infected with 15 MOI PR8 on ice for 1 h to synchronize virus attachment and then shifted to 37°C for 30 min to allow internalization of the adsorbed virions. The cells were washed twice, and the internalized virus was labeled with anti-influenza NP-antibody. IAV entry was significantly reduced by an average of 35% in SM depleted cells compared to untreated ones (Figures 3C,D). These results were confirmed by real-time PCR of the total viral RNA (data not shown). Therefore, SM is implicated during the entry step of IAV infection.

### ASMase Is Dispensable for IAV Infection

**FIGURE 4:**
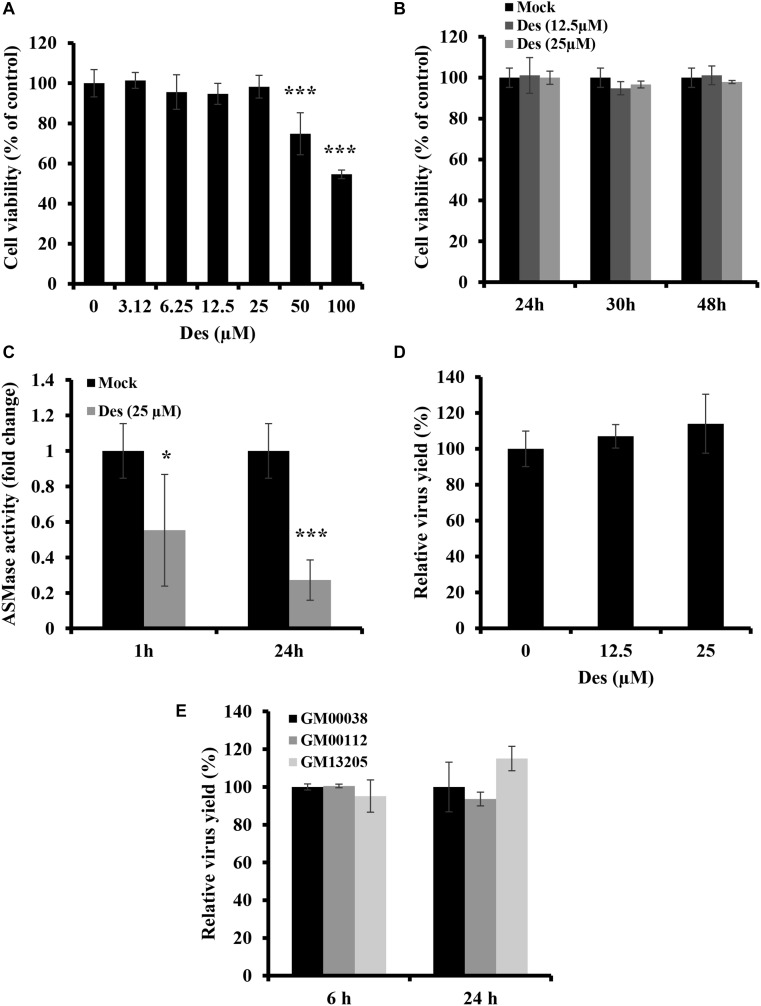
ASMase is dispensable for IAV infection **(A,B)** Cytotoxicity of desipramine (Des) in A549 cells was assessed using the MTT assay. A549 cells were treated with increasing desipramine concentrations for 24 h **(A)** or with desipramine at 12.5 μM or 25 μM for the indicated durations **(B)**. **(C)** The effect of desipramine on ASMase activity in A549 cells was determined. A549 cells were treated with the vehicle or 25 μM desipramine, and ASMase activity was then assessed at 1 and 24 h post-treatment using Amplex^®^ Red Sphingomyelinase Assay Kit. **(D)** The impact of ASMase inhibition on IAV infection was assessed using the plaque assay and plotted relative to the vehicle-treated cells. A549 cells were either treated with desipramine for 1 h or left untreated prior to inoculation with PR8 (1 MOI) for 1 h in the presence or absence of the inhibitor. Cells were then incubated with or without the drug for 24 hpi, and viral titers were determined using the plaque assay. **(E)** Primary human fibroblasts deficient in ASMase activity (GM00112 and GM13205) and normal human primary fibroblasts (GM00038) were infected with PR8 at 1 MOI for the indicated durations. Normal human primary fibroblasts were used as a control. The virus yield was determined using plaque assay and plotted relative to the control. The results are representative of two independent experiments. Statistical significance was measured using *t*-test (*correspond to a *P-*value < 0.05 and ***correspond to a *P-*value < 0.001).

Some viruses which utilize lipid rafts for entry require ASMase for efficient infection. However, although IAV utilizes lipid rafts for entry, SM depletion was found to restrict virus entry and infection, suggesting the ASMase activity is not favored by the virus. Therefore, we sought to determine the role of ASMase during IAV infection. We first assessed whether pharmacological ASMase inhibition would affect virus production. To do so, we utilized desipramine, a functional inhibitor of ASMase. Dose and time-dependent cytotoxicity of desipramine on A549 cells were first studied using MTT assay. Desipramine showed no cytotoxicity on A549 cells at concentrations of 25 μM and lower upon treatment for 24 h (Figure 4A) and up to 48 h (Figure 4B). We further verified that desipramine (25 μM) treatment significantly reduces ASMase activity in A549 cells (Figure 4C).

We next examined the effect of ASMase inhibition on IAV propagation. A549 cells were treated with desipramine for 1 h before inoculation with 1 MOI of PR8 and the inhibitor was maintained in the culture media during infection. Treatment with desipramine at 12.5 and 25 μM did not affect IAV yield at 24 hpi (Figure 4D). To eliminate the possibility that residual ASMase activity due to incomplete inhibition (Figure 4C) might be sufficient for efficient viral replication, we assessed IAV replication in NPA fibroblasts, which have total loss of ASMase activity. Two NPA cell-lines (GM00112 and GM13205) and normal fibroblasts (GM00038) were inoculated with PR8 at 1 MOI and virus progeny was quantified at 6 and 24 hpi. IAV efficiently replicated in NPA cells similar to normal fibroblasts (Figure 4E). These findings indicate that ASMase activity is not required for IAV infection.

### IAV Infection Restricts ASMase Activity at Later Time-Points Post-infection

**FIGURE 5:**
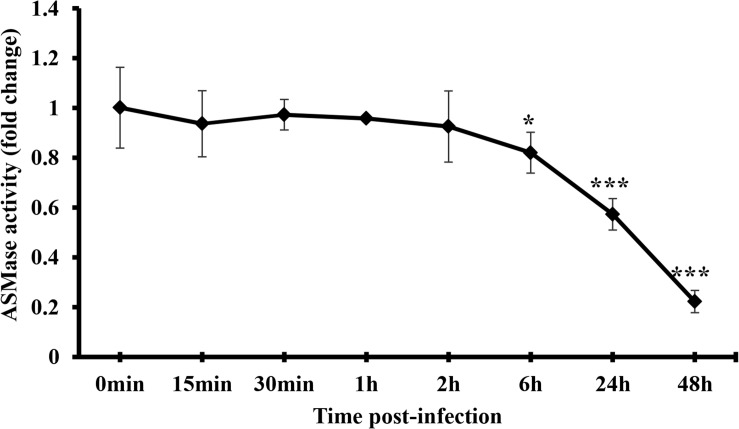
IAV infection restricts ASMase activity at later time-points post-infection. A549 cells were infected with PR8 at 1 MOI or left uninfected. Whole-cell lysates were used to assess ASMase activity at different durations post-infection using Amplex Red Sphingomyelinase Assay Kit. Results shown are fold changes of fluorescence in infected cells compared to their time-matching controls (uninfected cells). The average results of two independent experiments are shown. Statistical significance was measured between infected cells and their time-matching controls using *t*-test (*correspond to a *P-*value < 0.05 and ***correspond to a *P-*value < 0.001).

The finding that SM is required for IAV propagation and that ASMase activity is dispensable led us to investigate whether the virus suppresses ASMase activity to preserve the SM pools available in the host cell. Therefore, we measured ASMase enzymatic activity in control (non-infected) and IAV-infected A549 cells. The cells were infected with PR8 at 1 MOI, and ASMase activity was assayed at different time points up to 48 hpi. No detectable change in ASMase activity was observed at early time-points post-infection up to 2 h (Figure 5). However, a significant decrease in ASMase activity was observed at 6, 24, and 48 hpi, with only 20% residual enzymatic activity being detectable at 48 hpi. This observation indicated that IAV significantly inhibits ASMase activity at later stages of infection to possibly preserve SM for subsequent infection cycles.

### Virus SM Depletion Decreases IAV Infectivity

**FIGURE 6:**
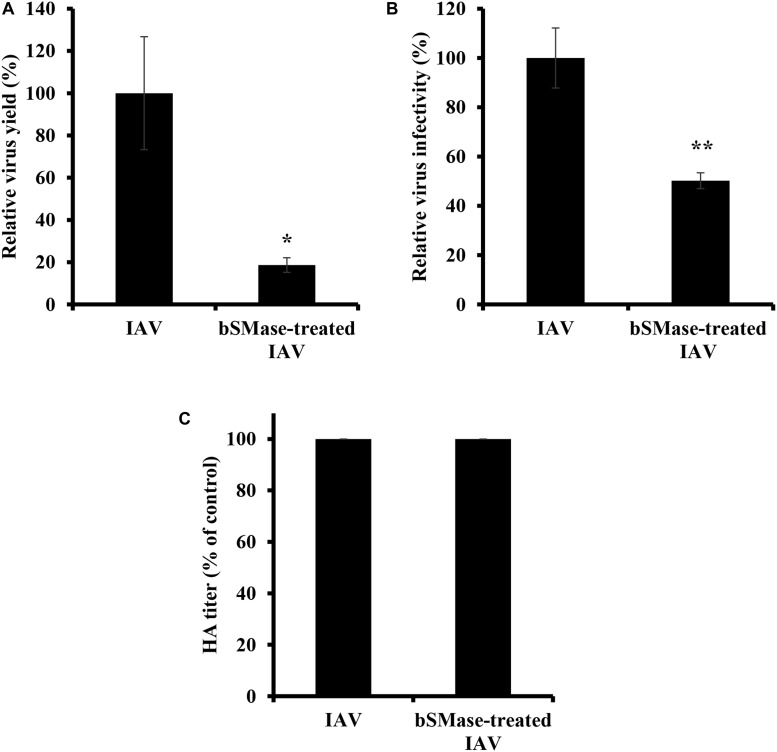
Virus SM depletion reduces IAV infectivity. The IAV PR8 stock was treated with 0.1 U/ml bSMase or with the vehicle (HBSS^+^) at 37°C for 1 h. **(A)** Treated or untreated PR8 was inoculated onto A549 cells after dilution in VIM to obtain an MOI of 1. Control cells were inoculated with untreated virus particles. Virus titers were assessed at 24 hpi using the plaque assay, and the virus yield was compared to control cells. **(B)** Alternatively, the infectivity of bSMase-treated or untreated IAV PR8 was assessed by plaque reduction assay using MDCK cells. Confluent monolayers of MDCK cells were inoculated with 90 PFU of bSMase-treated or untreated virus, and plaques were counted at 72 hpi. The % of infectivity was calculated relative to the control cells. Means ± SD are shown. **(C)** Hemagglutination assay was performed to assess the HA levels in the bSMase-treated virus. The results are representative of two independent experiments. Statistical significance between bSMase-treated and vehicle-treated IAV was assessed using the *t*-test (*corresponds to a *P-*value < 0.05, **corresponds to a *P*-value < 0.01).

The virus envelope shares similar properties as the plasma membrane. The observation that virus entry is suppressed in bSMase-treated cells led us to speculate that IAV infectivity could be also affected by perturbations in the lipid content, specifically SM, of its envelope. To address this, PR8 virus stock treated with bSMase (0.1 U/ml) or with the vehicle (HBSS^+^), was diluted to 1 MOI and used to inoculate A549 cells (Figure 6A). Interestingly, the infectivity of bSMase-treated IAV was significantly impaired compared to the untreated stock (Figure 6A). This finding was also confirmed using a plaque reduction assay in which we demonstrated a 50% reduction in the infectivity of the bSMase-treated virus (Figure 6B). To rule out the possibility that the reduction of infectivity of the bSMase-treated virus is due to the alteration in the HA protein density on the virus envelope, we assessed the hemagglutination ability of bSMase-treated virions as an indirect measure of the HA content. We found that hemagglutination titer did not change following treatment with 0.1 U/ml bSMase (Figure 6C), suggesting that the observed reduction in infectivity is not due to alteration of HA levels.

**FIGURE 7:**
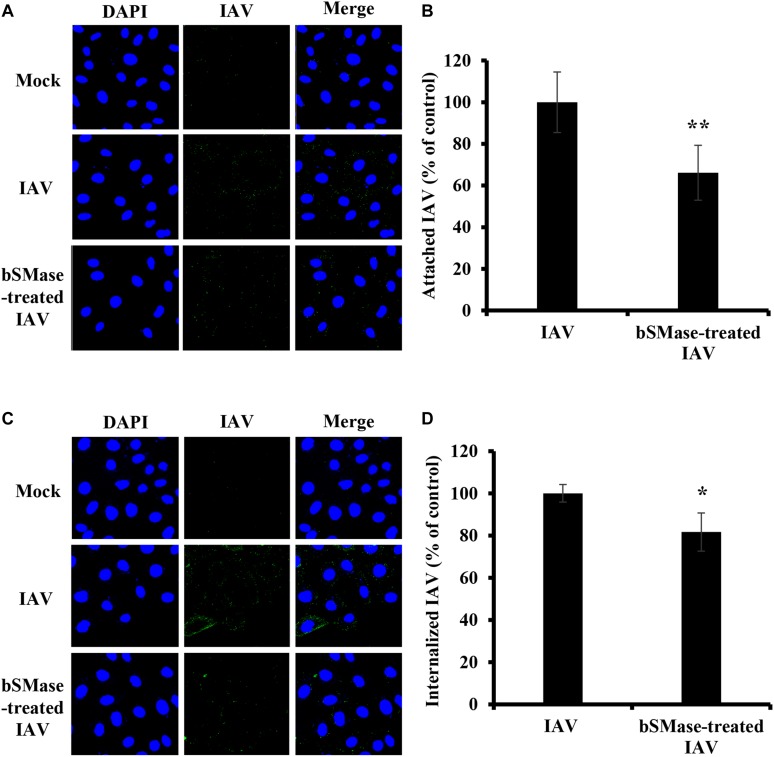
Virus SM depletion impairs attachment and entry. The IAV PR8 stock was treated with 0.1 U/ml bSMase (bSMase-treated IAV) or with the vehicle (HBSS^+^) (IAV) at 37°C for 1 h. **(A–D)** Treated or untreated PR8 was inoculated onto A549 cells after dilution in VIM to obtain an MOI of 15. Control cells were inoculated with untreated virus particles. Cells were infected for 1 h at 4°C to allow virus adsorption and then washed with cold PBS^+^. After adsorption, attached virions were labeled using mouse anti-influenza A (H1N1) antibody followed by Alexa Fluor 488 conjugated anti-mouse antibody (green) **(A,B)**. Alternatively, following viral adsorption, the cells were placed at 37°C for an additional 30 min to allow virus entry and internalized virions were assessed using mouse anti-NP antibody followed by Alexa Fluor 488 conjugated anti-mouse antibody (green) **(C,D)**. Mock cells were left untreated/uninfected and used to confirm the specificity of the antibodies. DAPI (blue) was used as a nuclear stain. The images were taken using confocal microscopy with a 63x oil objective lens **(A,C)**. The variation of fluorescence intensities was measured using ZEN software **(B,D)**. Means ± SD are shown. The results are the average of two independent experiments. Statistical significance between bSMase-treated and Mock-treated IAV was assessed using the *t*-test (*corresponds to a *P-*value < 0.05, **corresponds to a *P*-value < 0.01).

Next, we examined the effect of viral envelope SM depletion on IAV attachment and internalization. A549 cells were inoculated with bSMase- or vehicle-treated PR8 at 15 MOI at 4°C for 1 h. The binding of the SM-depleted virus was significantly reduced compared to the vehicle-treated one (Figures 7A,B). To assess the entry of the SM-depleted virus, the cells were incubated at 37°C for 30 min following adsorption. The bSMase-treated virus displayed reduced internalization compared to the vehicle-treated virus (Figures 7C,D). These findings indicate that viral envelope SM-depletion reduces both binding and internalization of IAV, resulting in suppressed infection.

## Discussion

Lipid rafts, membrane microdomains enriched in SM and cholesterol, were shown to be utilized during different phases of the IAV replication cycle (Rossman and Lamb, 2011). In addition, sphingolipids and their metabolizing enzymes were shown to have important roles during IAV life cycle (Hidari et al., 2006; Seo et al., 2013; Vijayan and Hahm, 2014; Soudani et al., 2019). However, the role of SM, a major sphingolipid component of the cell plasma membrane and endomembranes, during IAV infection, has not been fully explored. In this study, we demonstrated that efficient IAV infection is dependent on intact SM in the plasma membrane of the host cell and the viral envelope, specifically at the virus entry step, while ASMase activity seems to be dispensable.

To directly test for the requirement of intact membrane SM during IAV infection, we treated cells with exogenous bSMase before infection to deplete membrane SM. Exogenously applied bSMase, specifically hydrolyzes membrane SM into ceramide in the outer leaflet of the plasma membrane (Zhang et al., 1997). Depletion of SM within lipid rafts by bSMase was shown to impair multiplication of viruses that require SM during infection such as Ebola virus, Japanese encephalitis virus, Rubella virus, and porcine alphaherpesvirus (Miller et al., 2012; Taniguchi et al., 2016; Otsuki et al., 2018; Pastenkos et al., 2018). In line with these studies, we demonstrated that depleting plasma membrane SM by bSMase suppresses IAV infection. The reduction of virus replication upon bSMase treatment was observed both for laboratory H1N1 strain (PR8) and an H1N1pdm09 virus (Cal07), indicating that the effect of SM depletion is not strain-specific. Exogenous bSMase specifically hydrolyzes SM without affecting cholesterol levels within lipid rafts (Mizuno et al., 2011), which confirms that the reduction of IAV observed in bSMase-treated cells is explicitly attributed to the loss of SM. In our study, treatment with exogenous SM was found to enhance IAV replication. The modest enhancement of virus infection may be due to the already SM-saturated cellular membrane in A549 cells, as revealed by the lipidomic and sphingolipidomic analysis of A549 cells (He et al., 2016; Huang et al., 2018). This might explain the moderate increase in virus yield observed after SM treatment. Collectively, these results provide strong evidence for the requirement of SM during IAV infection.

Although lipid rafts were strongly associated with the assembly and budding of IAV, Eierhoff et al. (2010) showed that disruption of lipid rafts by MβCD impaired IAV internalization. In addition, lipid rafts are selected by IAV for binding and endocytosis into the host cells (Verma et al., 2018). It was previously demonstrated that HA-mediated fusion of influenza virus depends on the lipid composition of the host membrane (Chernomordik et al., 1997). In our experiments, cells were only treated with bSMase prior to IAV infection, and SM turnover from intracellular stores is rapid (Slotte et al., 1990). Thus, it is expected that SM is replenished by the time of virus budding. This indicates that, in addition to the previously reported role of intact SM pathway during IAV assembly and budding (Tafesse et al., 2013), SM is required during the early stages of IAV life cycle. Notably, the decrease in virus entry was partial, most likely due to the multiple internalization mechanisms utilized by IAV that may or may not involve lipid rafts such as clathrin-dependent endocytosis (CDE), clathrin independent endocytosis (CIE), caveolin independent endocytosis, macropinocytosis, and raft dependent endocytosis (Edinger et al., 2014; Verma et al., 2018).

Since some viruses that utilize lipid rafts during infection show a requirement for ASMase during infection (Steinhart et al., 1984; Grassme, 2005; Miller et al., 2012), we assessed the association between IAV infection and endogenous ASMase. We examined the effect of pharmacological ASMase-inhibition and ASMase-genetic deficiency on IAV infection. We found that, unlike Ebola virus, adenovirus, and rhinovirus (Grassme, 2005; Miller et al., 2012; Huang et al., 2018), ASMase inhibition using desipramine did not affect IAV infection. Furthermore, IAV was able to efficiently replicate in ASMase-deficient NPA cells. NPA cells have a mutation in the *SMPD1* gene that results in total loss of enzyme activity (Levade et al., 1986). Unlike rhinovirus which induces ASMase activation during infection (Grassme, 2005), ASMase was not activated in IAV-infected cells but was rather inhibited in a time-dependent manner. Consistent with our finding, Sindbis virus, which requires SM during infection, also inhibits ASMase in wild type-infected mice (Ng and Griffin, 2006). The decrease in ASMase activity observed during infection may be virus-induced to maintain the SM membrane pools required during the subsequent replication cycles or cell-induced to conserve SM needed for cellular activity. Together these results indicate that IAV does not require ASMase activity during infection.

Desipramine belongs to the group of lysosomotropic weak bases, which includes amantadine and chloroquine, both known to inhibit IAV (Wang et al., 1993; Di Trani et al., 2007; Kornhuber et al., 2010). These compounds diffuse through endosomal membranes and become protonated, thus increasing the endosomal pH and causing detachment and degradation of AMSase (Kornhuber et al., 2010). A common mechanism underlying the inhibitory effect of amantadine and chloroquine against IAV is through inhibiting endosomal activation and low-pH fusion of the virus. As such, this inhibitory effect varies according to the pH-dependency of the virus (Steinhauer et al., 1991; Di Trani et al., 2007). Besides, amantadine blocks the viral M2 channel and subsequently inhibits the uncoating step of the virus life cycle (Wang et al., 1993). In this study, desipramine had no inhibitory effect against IAV despite being a weak base. Desipramine is much less potent in neutralizing endosomal vesicles compared to chloroquine albeit having a higher inhibitory effect against ASMase (Kornhuber et al., 2010; Nadanaciva et al., 2011). Therefore, the lack of inhibitory effect by desipramine against IAV might be both due to its failure to increase the endosomal pH above the threshold required for activation of PR8 HA and the fact that ASMase is dispensable for its replication.

Sphingomyelin in the viral envelope was shown to be essential for some viruses. For example, depletion of SM from the envelope of hepatitis C virus and bovine herpesvirus-1 impaired their infectivity (Aizaki et al., 2008; Pastenkos et al., 2018). IAV acquires its envelope upon exiting the cell. Thus, the virus envelope shares similar properties as the plasma membrane. The envelope of IAV is mainly composed of host-derived lipid rafts that are selectively incorporated into the envelope and are highly enriched in SM, cholesterol, and phosphatidylcholine (Scheiffele et al., 1999). HA and NA viral glycoproteins preferentially concentrate in those lipid microdomains during assembly and budding (Leser and Lamb, 2005), suggesting that these glycoproteins also concentrate within the lipid rafts in the viral envelope. In our study, the depletion of SM in IAV envelope by bSMase treatment reduced virus infectivity without affecting the HA levels of the viral envelope. Both cell attachment and internalization of SM-depleted IAV were impaired. While we showed that the HA concentration is not changed upon the treatment of the virus with bSMase, it remains possible that the clustering of the envelope glycoproteins is affected due to the disruption of the lipid rafts. The clustering of HA protein within lipid microdomains was shown to be critical for efficient receptor binding and membrane fusion (Takeda et al., 2003). It is also possible that disrupting viral SM might interfere with the barrier properties of the envelope or the lipid mixing step proceeding membrane fusion. Supporting the latter hypothesis, bSMase treatment was shown to abolish rubella virus E1/E2-mediated membrane fusion (Otsuki et al., 2018).

In summary, our present work provides strong evidence that cellular and viral SM is required for efficient IAV infection, particularly during its attachment and entry into host cells. Therefore, we suggest that the previously reported co-localization of IAV with lipid rafts in the host membrane during internalization (Eierhoff et al., 2010; Verma et al., 2018) might be attributed to the SM content of these rafts. However, further investigations are needed to determine the precise mechanisms by which viral and cellular SM mediate IAV entry. Our findings provide support to targeting sphingolipids and SM in particular as an antiviral approach against IAV.

## Data Availability Statement

The raw data supporting the conclusions of this article will be made available by the authors, without undue reservation, to any qualified researcher.

## Author Contributions

HZ and AA designed the experiments, analyzed the data, and drafted the manuscript. GD helped with experimental design and data interpretation. AA performed the experiments. NS performed the confocal microscopy. All authors reviewed and approved the final manuscript.

## Conflict of Interest

The authors declare that the research was conducted in the absence of any commercial or financial relationships that could be construed as a potential conflict of interest.
